# Supplementary material for: Intraoperative alteration in the vital signs of diabetic patients during cataract surgery with local anesthesia
Source: PLoS One. 2022 Mar 22;17(3):e0265135. doi: 10.1371/journal.pone.0265135 (PMC8939796; doi:10.1371/journal.pone.0265135)
Supplement: Attachment — Submitted filename: Author response.docx [file pone.0265135.s002.docx]

Author response to the reviewers

We greatly appreciate the reviewers for many valuable comments given to our manuscript. We would provide the point-by-point answers for each comment. In addition, we would announce that the number of DM group has been changed from 104 to 103 after recounting the subjects and values of associated parameters have been slightly changed after recalculation, which does not affect the conclusion.

Reviewer #1: This study evaluated the intraoperative vital signs and associated factors under local anesthesia. I propose several points to be clarified.
1. The authors described the operation was performed within 30 mins. Please provide the mean operation time in table 1.

**Response:** Thank you very much for this comment. Firstly, we apologize for a mistyping of operation time. Actually, all surgeries were performed within 50 minutes (not 30 minutes) which has been corrected in the text (page 6, line 9). In addition, the mean operation times have been added in Table 1. In addition, vales of BMI and eGFR have also been presented in Table 1.

2. Were there any patients who needed additional medications due to high blood pressure before the surgery? If so, please describe that point in method section, and provide that information in result section such as the proportion of patients who were administered additional medication to control the high BP.

**Response:** We do not use additional hypotensive drugs before cataract surgery and rarely use such drugs during the surgery. If systolic BP of a patient is over 200 mmHg we firstly advise the patient to relax and repeat BP measurements several times (usually every 5 minutes). If BP is not decreased after repeated BP measurements, an injection of hypotensive drug might be used depends on surgeon’s discretion. Hence, it is quite rare to use additional hypotensive drugs within 15 minutes from the beginning of surgeries. However, we have mentioned about the possible use of hypotensive drugs as a limitation of this study in discussion part. (page 15, line 4-6)

3. Please provide the ocular information affecting the cataract surgery. Were all cases senile cataract? And if it is possible, axial length, presence of uveitis, or previous surgical history should be provided.

**Response:** Thank you for this comment. We agree that ocular status may affect cataract surgery and difficult procedures might cause intraoperative pain which could influence vital signs. However, in the present study, we obtained the data after anonymization of personal information (patient’s name, ID of clinical record etc.) and no correspondence table was made. Hence, we cannot obtain additional data about ocular status or past history.

4. The pulse rate and systolic BP can be affected by the pain sensation due to surgical procedure such as iris touching. The pupil of DM patients usually dilated poorly. If it is possible, please state the information about the pupil size or posterior synechia.

**Response:** We really appreciate the reviewer for this advice since we did not include intraoperative pain as a possible association factor with vital signs. Unfortunately, we cannot search additional information from anonymized data. Therefore, we have mentioned the possible influence of intraoperative pain as a limitation of this study in discussion part. (page 15, line 2-4)

5. In the same context as point 4, young patients usually feel pain better than older patients. Although, the mean age of two groups is not different significantly, DM patients were younger in table 1. I think presenting the factors which is not significant (including age) in table 2 and 3 would be helpful for better understanding of readers.

**Response:** We have presented all explanatory variables in Table 2 and 3 which should help the readers to understand non-significant factors.

Reviewer #2: The authors evaluated the intraoperative alteration of the vital signs in diabetic patients during cataract surgery with local anesthesia and emphasized the need for careful monitoring by the physicians to avoid possible systemic complications However, there are several concerns to be clarified before publication.

1. In figure 3, I think it is necessary to correct the figure legend as "Time course of pulse rate"

**Response:** Thank you very much for this comment. We have corrected the mistyping.

2. In figure 1, the mean SBP in DM group was more than 150 mmHg at 5-10 minutes from the beginning of surgery. Did you take any treatment for that?

**Response:** We rarely use additional hypotensive drugs during cataract surgery. If systolic BP of a patient is over 200 mmHg we firstly advise the patient to relax and repeat BP measurements several times (usually every 5 minutes). If BP is not decreased after repeated BP measurements, an injection of hypotensive drug might be used depends on surgeon’s discretion. Hence, it is quite rare to use additional hypotensive drugs within 15 minutes from the beginning of surgeries. However, we have mentioned about the possible use of hypotensive drugs as a limitation of this study in discussion part. (page 15, line 4-6)

3. In general, cataract surgery does not cause much pain, but blood pressure and pulse rate could be different depending on the severity of pain. It is better to describe whether you have considered this.

**Response:** We appreciate the reviewer for this advice. Actually, we did not evaluate intraoperative pain in this study. However, unfortunately, we cannot perform further evaluation for additional factors since the original data of present study were obtained after anonymization of personal information (patient’s name, ID of clinical record etc.) without correspondence table. Therefore, we have mentioned the possible influence of intraoperative pain as a limitation of this study in discussion part. (page 15, line 2-4)

4. There were several reports that bradycardia, hypotension associated lidocaine toxicity. Therefore, the possibility that lidocaine administration used for sub-tenon’s block may affect purse rate or blood pressure should be considered.

**Response:** Thank you for this comment. We agree that lidocaine may affect vital signs. In the present study, sub-Tenon’s lidocaine was administrated for both of DM patients and non-DM patients, so we consider that the difference between these two groups would remain under the possible influence of lidocaine.

Revised parts are indicated in red. We hope that all reviewers’ comments have been adequately addressed. Again, thank you so much for reviewing our manuscript.
